# An Elastin-like Polypeptide-fusion peptide targeting capsid-tegument interface as an antiviral against cytomegalovirus infection

**DOI:** 10.1038/s41598-024-60691-6

**Published:** 2024-05-04

**Authors:** Komal Beeton, Dipanwita Mitra, Adesanya A. Akinleye, John A. Howell, Christian S. Yu, Gene L. Bidwell, Ritesh Tandon

**Affiliations:** 1 Department of Cell and Molecular Biology, Center for Immunology and Microbial Research, University of Mississippi Medical Center, 2500 North State Street, Jackson, MS 39216 USA; 2 Department of Pharmacology and Toxicology, University of Mississippi Medical Center, 2500 North State Street, Jackson, MS 39216 USA; 3 Department of Neurology, University of Mississippi Medical Center, 2500 North State Street, Jackson, MS 39216 USA; 4 Office of Research Infrastructure Programs, National Institute of Health, 6701 Democracy Blvd., Bethesda, MD 20892 USA

**Keywords:** Drug discovery, Microbiology, Diseases

## Abstract

The tegument protein pp150 of Human Cytomegalovirus (HCMV) is known to be essential for the final stages of virus maturation and mediates its functions by interacting with capsid proteins. Our laboratory has previously identified the critical regions in pp150 important for pp150-capsid interactions and designed peptides similar in sequence to these regions, with a goal to competitively inhibit capsid maturation. Treatment with a specific peptide (PepCR2 or P10) targeted to pp150 conserved region 2 led to a significant reduction in murine CMV (MCMV) growth in cell culture, paving the way for in vivo testing in a mouse model of CMV infection. However, the general pharmacokinetic parameters of peptides, including rapid degradation and limited tissue and cell membrane permeability, pose a challenge to their successful use in vivo. Therefore, we designed a biopolymer-stabilized elastin-like polypeptide (ELP) fusion construct (ELP-P10) to enhance the bioavailability of P10. Antiviral efficacy and cytotoxic effects of ELP-P10 were studied in cell culture, and pharmacokinetics, biodistribution, and antiviral efficacy were studied in a mouse model of CMV infection. ELP-P10 maintained significant antiviral activity in cell culture, and this conjugation significantly enhanced P10 bioavailability in mouse tissues. The fluorescently labeled ELP-P10 accumulated to higher levels in mouse liver and kidneys as compared to the unconjugated P10. Moreover, viral titers from vital organs of MCMV-infected mice indicated a significant reduction of virus load upon ELP-P10 treatment. Therefore, ELP-P10 has the potential to be developed into an effective antiviral against CMV infection.

## Introduction

Human Cytomegalovirus (HCMV) is a ubiquitous betaherpesvirus infecting 30–100% of the population worldwide^1^. Although HCMV can infect multiple tissue types, it primarily infects monocytes, lymphocytes and epithelial cells^1,2^. Similar to other herpesviruses, HCMV establishes latency and continues to exist in a dormant form within the host's body throughout its lifetime. HCMV can spread through various routes, including saliva, urine, sexual transmission, and organ transplantation^3^. While HCMV infection is largely asymptomatic in immunocompetent individuals, it can cause major problems in immunocompromised individuals such as AIDS patients and organ transplant recipients^4,5^. Additionally, HCMV is one of the most common infectious causes of congenital birth defects in the United States, leading to hepatosplenomegaly, sensorineural hearing loss, cognitive delay, and fetal mortality^6–8^. Currently, there is no licensed vaccine for HCMV, and the available antivirals such as ganciclovir, valganciclovir and foscarnet have limited bioavailability, efficacy, and notable side effects^6,9^. Consequently, the development of novel antiviral agents that can effectively combat HCMV infection with improved safety profiles is urgently required.

The infectious virus particle of HCMV consists of a 230 Kb linear dsDNA genome embedded within an icosahedral nucleocapsid, surrounded by a proteinaceous layer called tegument which is further enclosed by a host-derived lipid envelope containing viral glycoproteins^9,10^. The proteins in the tegument layer are fully formed and released into the cell upon virus entry. They play an important role in virus replication, gene expression, immune evasion, and virion maturation during the later stages of infection^11–13^. The maturation of HCMV occurs in two different compartments, the host cell nucleus and cytoplasm^14^. Replication of the viral genome occurs in the host cell nucleus, followed by capsid assembly and subsequent encapsidation^15^. Once the nucleocapsids are assembled, they traverse out of the host nucleus via a complicated process of nuclear egress involving primary envelopment and de-envelopment and localize in the cytoplasm where they accumulate in a Golgi-derived juxtanuclear body known as viral cytoplasmic assembly compartment (vAC)^16,17^. The vAC consists of various viral structural and tegument proteins that are imperative for secondary maturation^16^. One such tegument protein, pp150 (basic phosphoprotein 150), encoded by the UL32 gene is specifically known to be essential for the final stages of virus maturation^18,19^. pp150 is expressed late in infection and is the second most abundant tegument protein found in HCMV^11^. It is known to interact with the capsid proteins selectively through its amino-terminal region during tegument assembly^20^. The main function of pp150 is to stabilize and maintain the organization of the nucleocapsid during its secondary envelopment within the vAC^15,18,19^. Structurally, pp150 forms upper and lower helix bundles connected by a central long helix. The N-terminal one-third of pp150 (pp150nt) has conserved regions, including a cysteine tetrad and two betaherpesvirus conserved regions (CR1 and CR2) that are present at the binding interface of pp150 with capsid proteins. The pp150nt-capsid interaction occurs in both upper and lower helix bundles. The atomic model suggests that N-terminal residues 1 to 275 alone are sufficient for the pp150-capsid binding^21,22^. These atomic details have paved the way to explore pp150 as a target for the development of new antivirals.

Previously, our laboratory designed peptides similar in sequence to pp150 conserved regions (CR1, CR2 and cysteine tetrad). The goal was to competitively inhibit pp150-capsid interaction and hence viral maturation^23^. Our study established that of all the tested peptides, a peptide targeted to pp150 conserved region 2 (PepCR2 or P10) led to a significant reduction in both HCMV and MCMV growth as well as spread in cell culture. P10 treatment rendered pp150 sequestered in the nucleus of the infected cells as visualized by immunofluorescence microscopy. This is most likely due to the disruption of pp150 loading onto nucleocapsids in the nucleus, and a resultant compromise in nuclear egress as well as later steps in virus maturation including the successful organization of the vAC. These data correlate with a proposed mechanism where pp150-peptides would interfere with virus maturation and ultimately inhibit the virus growth^23^.

Peptide therapeutics have emerged as a promising new strategy for targeted therapy in various diseases including cancer, metabolic disorders, and viral infections. Antiviral peptides are widely used as an effective treatment for inhibiting viral infections caused by respiratory viruses, HIV, and others^24^. Peptide-based therapeutics offer several advantages over conventional small molecule drugs, such as high specificity and selectivity for the target, and lower incidence of side effects^25,26^. However, the clinical translation of peptides is hindered by several challenges. When applied in vivo, they are rapidly degraded and exhibit poor pharmacokinetic properties. Furthermore, their often-charged nature can limit their ability to penetrate the cell membranes, thus hindering their effectiveness. To overcome these limitations, our lab’s approach involves conjugating peptides with carrier molecules, such as elastin-like polypeptide (ELP), to improve their pharmacokinetic and pharmacodynamic properties^27,28^. ELP is a synthetic protein derived from mammalian tropoelastin. ELP is composed of a repeat motif, most commonly valine-proline-glycine-x-glycine (VPGxG)n where x can be any amino acid other than proline and n is the number of pentapeptide repeats^27^. ELPs have been used for the delivery of therapeutic peptides in several disease applications^29–35^. ELPs possess several desirable characteristics, such as biocompatibility, non-immunogenicity, prolonged plasma half-life, and reversible phase transition behavior^26,28^. This unique phase transition property enables the purification of ELPs and ELP-fusion proteins through a process called inverse transition cycling, eliminating the need for chromatographic purification methods^28^. Moreover, ELP size can be modified by changing the number of VPGxG repeats, and the “x” residue can be substituted, to tune the transition temperature^28^. Therefore, due to these numerous benefits of ELP, we conjugated the P10 peptide with ELP, which serves as a suitable carrier molecule for improved delivery and efficacy of the therapeutic peptide. We hypothesize that the fusion of the P10 peptide with ELP will enhance bioavailability and antiviral activity against cytomegalovirus infection. The study of HCMV is limited by its species-specificity, precluding the use of animal models, including non-human primates. As the amino-terminal one-third of pp150 contains CR1 and CR2, which are known to be the two most highly conserved regions among all beta herpesvirus pp150s^20^, we utilized MCMV for in vivo studies. Our data show that ELP-conjugated peptide successfully inhibits MCMV growth in vitro. Our findings suggest that conjugating ELP with P10 enhances the bioavailability and inhibitory potential in vivo, making it a promising candidate for the development of an effective antiviral therapy against CMV infection.

## Results

### Generation of ELP-P10 chimeric fusion protein

**Figure 1:**
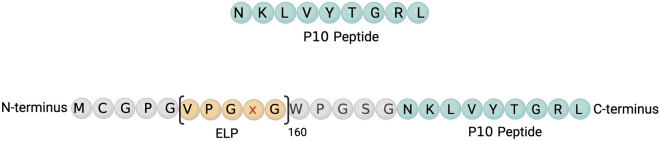
Illustration of the peptide constructs: the unconjugated P10 peptide (top) and ELP-conjugated P10 peptide (bottom) with the linker sequences shown in grey. ELP-P10 chimeric fusion construct was constructed by conjugating the P10 peptide to the C terminal of ELP.

To overcome the limitations of using free peptides in an in vivo system, we conjugated P10 with ELP. The ELP used in our study has a size of ~ 62-kDa with 160 repeats and a phase transition temperature of 65 °C. The ELP-P10 chimeric fusion protein was constructed by appending the P10 peptide to the carboxyl (C) terminus of the ELP, while preserving the intact amino (N) terminal region of the ELP for subsequent rhodamine labeling (Fig. 1).

### ELP-P10 treatment inhibits MCMV growth in cell culture

**Figure 2:**
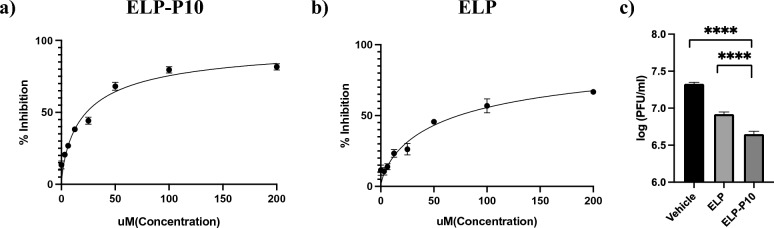
(**a**, **b**) Determining the Half maximal inhibitory concentration (IC_50_) of ELP-10 and ELP. MEFs were pretreated with different concentrations of ELP-P10 or ELP respectively in triplicates and then infected with MCMV at low MOI of 0.01. Cells were harvested 3 dpi, and viral titers were measured by plaque assay. The percentage inhibition of virus growth is plotted against different concentrations of ELP-P10 and ELP respectively. Standard error of mean (SEM) of ELP-P10 and ELP were plotted as error bars, and data analysis was done by one-way ANOVA, asterik represents p value < 0.0001 (**c**) Inhibition of MCMV growth with ELP-P10 and ELP. MEFs were pretreated with 80 μM of ELP-P10, ELP or saline (vehicle) in triplicates for 1 h and then infected with MCMV at high MOI of 3.0. Cells were harvested at 3 dpi and viral titers were assessed by plaque assay. SEM of inhibition by ELP-P10 and ELP from three different experiments were plotted as error bars using graphpad prism and data analysis was done using t-test, asterik represents p < 0.0001.

To test the inhibitory potential of ELP-P10 in an in vivo mouse model for future experiments, we first investigated the inhibitory potential of ELP-P10 against MCMV in cell culture. Since CMV is species-specific, we performed the MCMV experiments in mouse embryonic fibroblasts (MEFs). The inhibitory potential of the protein biopolymer–delivered peptide, ELP-P10 and ELP (control), was determined by pretreating MEFs with ELP-P10 or ELP with a concentration range of 1.5–200 µM or vehicle-treatment for 1 h and then infecting with MCMV at a low MOI of 0.01. Cells were harvested at 3 dpi, and virus yield was measured by counting the number of plaques using plaque assay. The half maximal inhibitory concentration (IC_50_) of ELP-P10 was calculated to be 23.41 μM (with a 95% confidence interval of 19.31 to 28.31 μM), and 73.24 μM for ELP (with a 95% confidence interval of 61.15–89.42 μM) (Fig. 2a, b). To determine the inhibitory potential of ELP and ELP-P10 after a greater viral challenge, MEFs were pretreated with ELP-P10 or ELP control before infecting them with MCMV at an MOI of 3.0. ELP-P10 and the ELP control were tested at ELP-P10’s IC_75_ of 80 μM for the high MOI experiment. The titers indicate a > ten-fold reduction in virus growth in ELP-P10 treated cells compared to the vehicle treatment group (Fig. 2c). Although ELP-control treatment showed inhibition of virus growth in infected MEFs, the viral titers in ELP-P10 treated cells were significantly lower compared to ELP-control treated cells.

**Figure 3:**
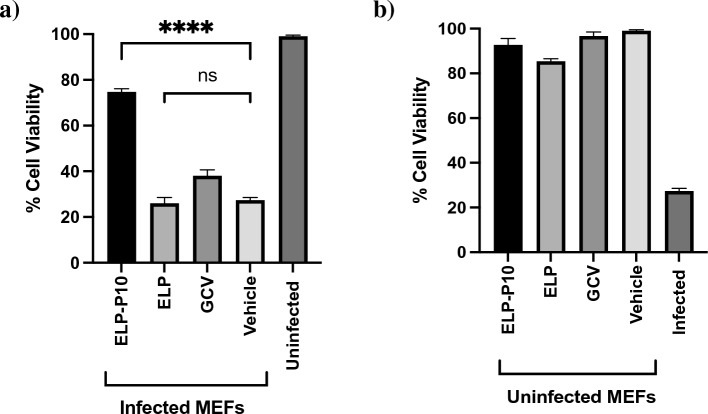
Cell Viability (%) in (**a**) infected MEFs (**b**) uninfected MEFs with different treatments groups. MEFs were pretreated with ELP and ELP-P10, and GCV (control) or vehicle-treated for 1 h in triplicates, and then infected with a high MOI of 3.0 with MCMV K181 or vehicle-infected. Cells were harvested at 3 dpi, and cell viability was assessed by trypan blue exclusion assay for both uninfected and infected groups. The standard error of the mean from three different experiments were plotted as error bars. Data were analyzed in GraphPad Prism with one-way ANOVA and showed significant differences (p < 0.05) between groups.

Next, to rule out any cytotoxic effects of ELP-P10 and to determine whether it could protect cells from CMV infection, a cell viability assay was performed on ELP-P10-treated infected and uninfected MEFs along with ELP-control and ganciclovir (GCV), an FDA-approved drug for CMV, as an appropriate inhibitory control. Following CMV infection, ELP-P10-treated cells had higher cell viability compared to control groups, indicating that ELP-P10 efficiently protected the MCMV-infected cells from virus-induced lytic cell death (Fig. 3a). Importantly, when administered at equimolar concentrations, ELP-P10 treatment resulted in a more efficient protection of cell viability than ELP control or the comparator antiviral drug GCV. To rule out any direct cytotoxic effects of ELP-P10, ELP, or GCV, cell viability was also determined in uninfected fibroblasts. Viability was not affected by ELP-P10, ELP control, or GCV at the concentrations used in the infection assay (Fig. 3b). To corroborate these findings, a metabolic assay was performed to look at the metabolitc activity of the cells following ELP-P10 and ELP treatment using CellTiter 96® AQueous One Solution Cell Proliferation Assay (MTS). The results largely mirrored the original results using crystal violet (Supplementary Fig. S1). ELP-P10-treated infected cells had higher cell viability compared with control groups, indicating that ELP-P10 protected the MCMV-infected cells from virus-induced lytic cell death (Supplementary Fig. S1a). There was little effect of any of the treatments in uninfected cells. ELP-P10 treatment led to 30% reduction in the viability of the uninfected cells. Although ELP-P10 reduced metabolic activity in the uninfected cells, it still showed a significant protective effect from MCMV infection (Supplementary Fig. S1b). Altogether, this demonstrates the potent antiviral properties of ELP-P10, indicating that the increased cell viability is not merely a non-specific effect of the carrier molecule (ELP) but rather a specific characteristic or mechanism associated with ELP-P10 against MCMV. Overall, these results indicate that ELP-P10 shows inhibitory potential against MCMV and could protect cells from lytic death associated with CMV infection.

### ELP conjugation of P10 enhances its bioavailability and accumulation in mouse organs

**Figure 4:**
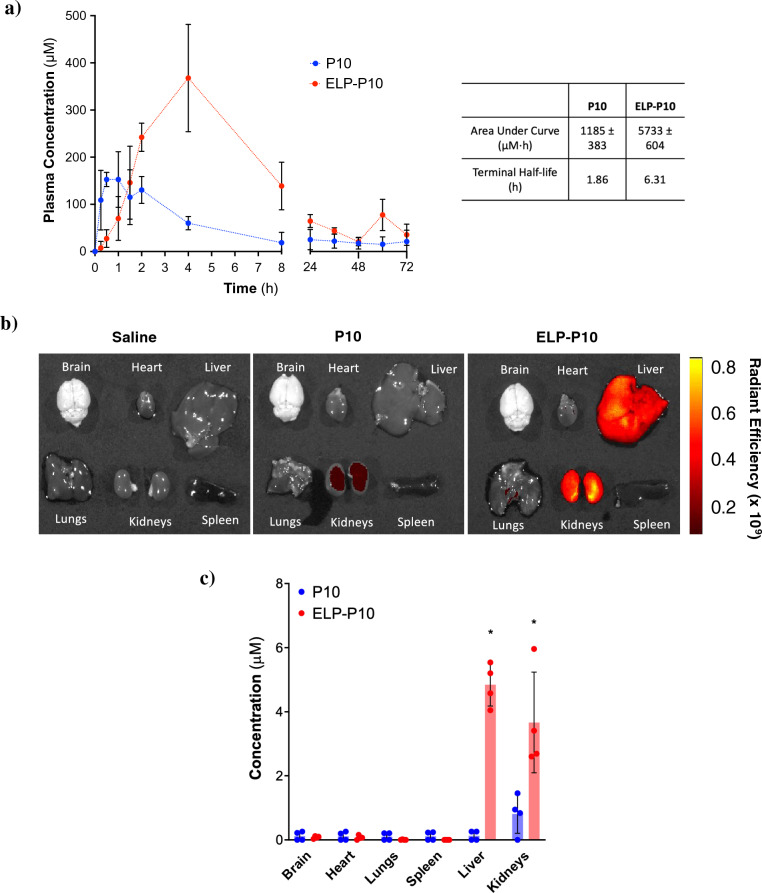
Pharmacokinetics and biodistribution of P10 and ELP-P10. Rhodamine-labeled P10 (free peptide) or ELP-P10 were administered by subcutaneous injection in mice (females, n = 4 per group). Plasma pharmacokinetics (**a**) were determined for 72 h after injection. In a separate cohort of mice (females, n = 4 per group), major organ biodistribution was determined 4 h after injection by ex vivo whole-organ fluorescence imaging (**b** and **c**). The standard deviations were plotted as error bars.

To test our hypothesis that ELP-conjugation to P10 will enhance its bioavailability, pharmacokinetic and biodistribution analysis of rhodamine-labeled P10 and ELP-P10 was performed to determine their plasma half-life and organ-specific accumulation. Upon subcutaneous injection of rhodamine-labeled P10 or ELP-P10, direct measurement of plasma fluorescence revealed that P10 displayed a rapid clearance from plasma compared to ELP-P10, which exhibited a significantly higher concentration in plasma (Fig. 4a). Notably, while P10 began to clear from plasma after 2 h, ELP-P10 demonstrated a peak concentration at 4 h, followed by a gradual decline. The area under the curve (AUC) for ELP-P10 was found to be 5 times greater than that of P10, indicating significantly higher bioavailability^36^ for ELP-P10. Moreover, fitting the clearance phase to a two-compartment pharmacokinetic model revealed that ELP-P10 had a terminal half-life of approximately 6.31 h compared to an approximately 1.9 h half-life for the free peptide.

For biodistribution, mice were subcutaneously injected with ELP-P10, P10, or vehicle control (saline), and vital organs were harvested 4 h post-injection. Interestingly, the whole organ ex vivo imaging of harvested organs demonstrated a very high accumulation of ELP-P10 in the kidneys, significant accumulation in the liver, and low levels in the other major organs. The unconjugated P10 peptide, however, accumulated at much lower levels than ELP-P10 in the kidneys and liver (Fig. 4b, c). These results collectively suggest that conjugation of P10 with ELP enhanced its bioavailability and accumulation in the liver and kidneys, suggesting its potential to be used as a therapeutic. Given that CMV is a major opportunistic infection in liver and kidney transplant patients^37^, the accumulation of the therapeutic in these organs suggests its potential to be used in transplant settings to reduce CMV infection.

### ELP-P10 treatment leads to a reduction in virus titers in MCMV-infected mouse organs

**Figure 5:**
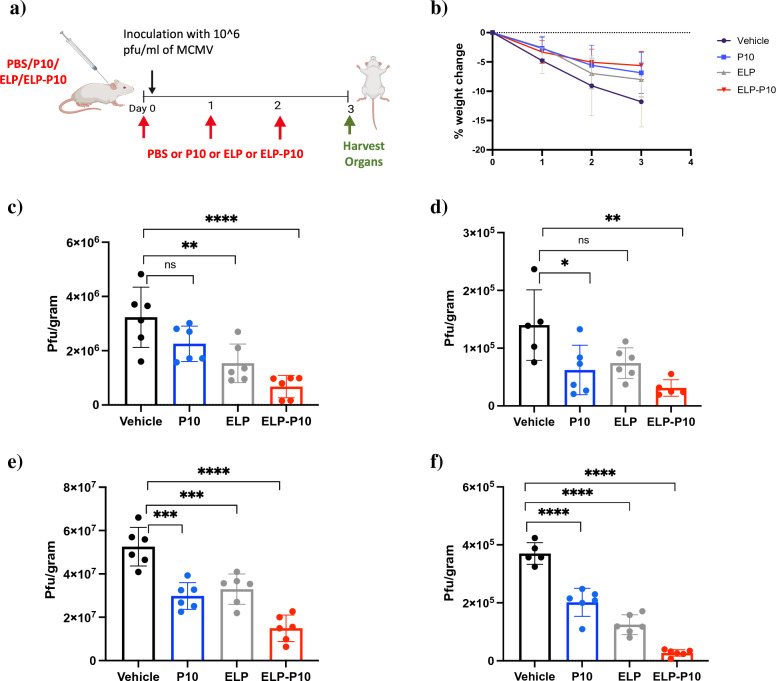
Efficacy of P10 (free peptide) and ELP-P10 in MCMV-infected mice: (**a**) Schematic of experimental setup. Mice (BALB/c, 3 males and 3 females per treatment group) were administered with 1000 nmol/Kg of P10 or ELP or ELP-P10 or vehicle subcutaneously followed by intraperitoneal infection with 10^6^ pfu/mL of MCMV (K181 strain) one-hour post treatment. Mice were sacrificed on day 4 post-infection and vital organs were harvested for titering. (**b**) Percentage change in body weight of animals with different treatment groups. Viral load in MCMV infected mouse organs (**c**) Liver, (**d**) Kidneys, (**e**) Spleen and (**f**) Heart with different treatments assessed by plaque assay. Data were analyzed in GraphPad Prism with one-way ANOVA and post-hoc Tukey’s multiple comparisons, asterisks represent p-value < 0.05. The standard deviations were plotted as error bars.

We further investigated the efficacy of P10 and ELP-P10 in a mouse model of CMV infection. Mice were subcutaneously injected with 1000 nmol/Kg of P10, ELP, ELP-P10, or vehicle (saline) and infected with a high dose (10^6^ Pfu total) of MCMV (K181 strain) intraperitoneally, one hour post-treatment. Mice were treated with the same dose of test agents every 24 h (based on pharmacokinetic data) and euthanized on day 4 to harvest the vital organs (liver, kidneys, heart, and spleen) (Fig. 5a). All the mice survived for the duration of the experiment. The infected but vehicle-treated group exhibited the highest percentage loss in the body weight (Fig. 5b). In concert with the loss in body weight, we also observed hunched behavior, ruffled fur, and reduced activity in the vehicle-treated group. In contrast, mice treated with ELP-P10, P10 peptide or ELP lost less weight following MCMV infection and did not show hunched behavior, ruffled fur, or reduced activity.

The vital organs of all animals were harvested at the end of the experiment, and viral titer was determined. These data indicated that a maximum reduction in viral titer resulted from ELP-P10 treatment compared to other treatment groups in all the vital organs (Fig. 5c–f). Viral titer was reduced by 79% in the liver (Fig. 5c), 77% in the kidney (Fig. 5d), 71% in the spleen (Fig. 5e), and 90% in the heart (Fig. 5f) following ELP-P10 treatment. Interestingly, ELP control showed significant but less potent reduction of viral titer in the liver, spleen, and heart, and a trend for reduction of titer in the kidney, which was consistent with earlier in vitro experiments demonstrating antiviral activity of the ELP carrier itself. The unconjugated P10 showed a significant reduction in virus titer compared to the control (PBS) treatment in the kidney, heart, and spleen, and a minor reduction in the liver that did not reach statistical significance. Overall, our results show that ELP-P10 has enhanced efficacy compared to P10 and ELP control. These findings suggest that the conjugation of P10 with ELP enhanced its efficacy to reduce viral load in a mouse model of CMV infection.

## Discussion

In this study, we demonstrated that ELP-P10 effectively inhibits the growth of MCMV in cell culture and in an in vivo model of CMV infection. A previous study from our lab has established that the CR2 region of HCMV tegument protein pp150 is amenable to targeting by sequence-specific peptide P10^23^. We used ELP as a suitable conjugate and carrier system to enhance the bioavailability of the selected peptide for future applications in an in vivo system.

We first sought to determine if the P10 peptide still retains its inhibitory potential when fused to the ELP carrier. As shown in Fig. 2c, ELP-P10 successfully inhibited MCMV growth in cell culture. This demonstrates that using ELP as a carrier did not interfere with the ability of P10 to disrupt viral infection, though it did lower the potency of the inhibitory peptide^23^. Ongoing work is exploring strategies to modify the ELP carrier or increase cellular uptake of the construct to increase potency. Interestingly, ELP itself showed a significant reduction in the virus growth. However, the IC_50_ of ELP was ~ three-fold higher compared to ELP-P10, and virus inhibition was observed only with the higher concentrations of ELP (Fig. 2a, b). One potential explanation for the non-specific inhibition by ELP is that the large size of the ELP molecule may hinder the interaction of viral proteins during the assembly of the virus. This interference caused by ELP might result in the non-specific inhibition of the virus growth. Another explanation could be the effects of large doses of ELP on cellular endocytosis/exocytosis processes. However, these hypotheses require further research to confirm their validity and to investigate the potential mechanism underlying the observed inhibitory effects of ELP.

To further strengthen our hypothesis that ELP conjugation of P10 will enhance its plasma life, we performed pharmacokinetic and biodistribution analysis with rhodamine-labeled P10 and ELP-P10. As expected, we observed an increase in the plasma half-life and bioavailability of ELP-P10 compared to the free P10 peptide, as the calculated area under the curve for ELP-P10 was five-fold higher compared to P10. Moreover, there was an increased accumulation of ELP-P10 in the mouse organs, specifically in the liver and kidneys. Higher accumulation of ELP-P10 in the liver and kidneys could be attributed to the unique properties of the ELP system. ELPs have an increased half-life and tissue accumulation compared to small peptides which are rapidly filtered by kidneys and cleared from the body more quickly. Due to the larger size of ELPs, their renal filtration is slower than that of free peptides, and they exchange between plasma and tissues more slowly. Therefore ELP-fused peptides accumulate for longer periods of time and at higher levels in organs like the liver and kidneys^30^. This contrasts with the free peptide P10 which is rapidly filtered by the kidneys and thus accumulates in kidneys to a very low level. Additionally, enhanced accumulation of ELP-P10 in the liver could be due to uptake by the reticuloendothelial system.

Further, to investigate if the enhanced bioavailability of ELP-P10 translated to enhanced antiviral efficacy, we tested the ability of ELP-P10 to inhibit virus growth in an in vivo murine model of MCMV infection. The observed percentage change in body weight in the ELP-P10 treated group was less compared to the untreated control group. This suggests a protective effect of ELP-P10 treatment. Moreover, the vehicle-treated (PBS) animals had severe signs of disease such as ruffled fur and hunched behavior compared to the P10, ELP, and ELP-P10 treatment groups. Previous studies on MCMV using a mouse model have shown that intraperitoneal administration of MCMV can cause significant pathology to the host and leads to high viral load in major organs, with maximum viral load in the liver and spleen as these are considered the principal sites of early viral replication^38–40^. Similarly, we observed high viral load in the liver and spleen of the vehicle treated mice. Notably, the viral titers from all infected vital organs were reduced by 80–90% in the ELP-P10 treated group, compared with less dramatic reductions in the P10 and ELP control groups. This could be attributed to the fact that ELP conjugation of P10 enhanced its plasma half-life and tissue accumulation. This can result in a more sustained concentration of the therapeutic in the infected organs, which can potentially lead to more inhibition and antiviral efficacy against MCMV. Despite the high viral load observed in the liver of infected mice (vehicle treatment), previous studies have shown that the virus production in liver does not appear to contribute to systemic viral spread^41,42^. CMV is a major opportunistic infection in the liver and kidneys and causes hepatitis and major problems in kidneys of transplant patients^37^. Therefore, the accumulation of the therapeutic in these organs suggests its potential to be used in transplant settings to reduce CMV infection.

Altogether, our findings represent an interesting proof of principle in demonstrating the inhibition of MCMV growth by a peptide targeting a non-surface viral protein. While ELP-P10 holds the potential to be developed into an effective therapeutic against cytomegalovirus infection, it is crucial to acknowledge that the calculated IC_50_ of ELP-P10 is several log steps higher than the approved antivirals for use in clinical applications. For instance, Enfuvirtide—an FDA approved antiviral for the treatment of HIV, has IC_50_ in the nanomolar range; Myrcludex B for treatment of Hepatitis B and D has demonstrated efficacy in picomolar concentrations^43,44^ These antivirals underscore the significance of achieving antiviral activity at lower dosage. Of note, the IC_50_ of unconjugated P10 is 1.3 µM, however, on conjugation with ELP there is almost a 20-fold increase in the IC_50_^23^_._ While a high IC_50_ is a concern, the IC_50_ is not the only parameter that determines the clinical viability of a novel therapeutic. The potential advantages conferred by ELP modification, such as prolonged plasma half-life and increased accumulation in the organs, plus the ease of producing ELP-P10 in gram quantities by recombinant production methods, may outweigh the cost of a higher IC_50_ of ELP-P10. Overall, our study indicates that ELP-P10 inhibits MCMV growth in vitro and in vivo. Therefore, it can serve as a potential antiviral for combating CMV infection.

## Methods

### Cells

Mouse embryonic fibroblasts (MEFs) were grown in Dulbecco’s Modification of Eagle’s Medium with 4.5 g/L glucose, l-glutamine and 1 mM sodium pyruvate (DMEM, Corning, catalog number 10-013-CM) containing 10% fetal bovine serum (R&D Systems Biotechne, Flowery Branch, GA, catalog# S11150H), 2 mM l-glutamine and 100 U/mL penicillin–streptomycin (Corning, Manassas, VA; catalog# 30-002-CI) at 37 °C with 5% CO_2_.

### Virus

MEFs were used to culture the K181 strain of MCMV. To prepare the virus stock, the MCMV was mixed with 3× autoclaved milk, which was derived from Carnation (Nestle) instant nonfat dry milk powder. The mixture was sonicated three times for 10 s each, with a 30-s gap in between, and then stored at − 80 °C. Additionally, 10% milk was prepared in nanopure water, with a pH of 7.0, and autoclaved three times.

### Generation of the ELP-P10 construct

A synthetic DNA cassette encoding the P10 sequence was synthesized with codons that were optimized for expression in *E. coli* (Life Technologies). The cassette was inserted into the pET25b+ expression vector between NdeI and BamHI restriction sites, with an SfiI site at the N-terminus of the P10 coding sequence. DNA containing the ELP coding sequence was excised from pUC19-ELP and cloned into the SfiI site, generating an in-frame fusion of ELP and P10. The ELP sequence contained 160 VPGxG repeats in which the x residue was V, G, or A in a 1:7:8 ratio. All constructs were confirmed by DNA sequencing.

### Purification of ELP-P10

The pET25b+ vector containing the ELP-P10 coding sequence was transformed into *E. coli* BLR(DE3) and selected on ampicillin agar plates. Liquid cultures (10 mL Terrific Broth (TB Dry, MP Biomedicals)) were inoculated and grown overnight, then used to inoculate 500 mL cultures that were grown for 16–20 h in 2 L shaker flasks. The pET system can produce low-level recombinant protein expression even without induction. Bacteria were harvested by centrifugation, lysed by sonication, and nucleic acids were precipitated with polyethyleneimine (PEI) and removed by centrifugation. The salt concentration of the soluble lysate was raised to approximately 200 mg/mL by the addition of NaCl, and the solution was heated at 50 °C until the ELP-P10 precipitated (as judged by the formation of a visibly cloudy solution). The precipitated ELP-P10 was collected by centrifugation, and the protein pellet was re-dissolved in cold PBS and centrifuged at 4 °C to remove any undissolved precipitate. This heat cycling process was repeated 3–5 times until purified protein was obtained. Purity was assessed by SDS-PAGE and the concentration of ELP-P10 and ELP was determined spectrophotometrically by measuring absorbance at 280 nm and utilizing the theoretical extinction coefficient of 6990 and 5500/M cm respectively, which was calculated from the protein’s primary sequence using the Expasy Protparam tool.

### IC_50_

MEFs were plated in 24-well tissue culture plates and grown to confluency. Cells were then pretreated with different concentrations (1.5 μM, 3.125 μM, 6.25 μM, 12.5 μM, 25 μM, 50 μM, 100 μM, 200 μM) of ELP-P10 or ELP in triplicates and then infected with MCMV at low MOI (multiplicity of infection) of 0.01. Cells were harvested 3 dpi (days post-infection), and viral titers were measured by plaque assay. The experiment was repeated 3 times to confirm reproducibility of data. The data were normalized in GraphPad Prism 9.0, using the number of plaques in the vehicle wells as 0% inhibition and the number ‘0’ as 100% inhibition. The percentage (%) inhibition was plotted against the concentration range.

### Inhibition of virus growth

MEFs were plated in 12-well tissue culture plates and grown to confluency in triplicates followed by treatment with appropriate concentrations of ELP-P10, ELP (equivalent to 10 µM of unconjugated P10) or vehicle (PBS) treated for 1 h and then infected with MCMV at high MOI of 3.0. Cells were harvested 3 dpi, and viral titers were assessed by plaque assay.

### Cell viability

MEFs were plated in 24-well tissue-culture plates and grown to confluency in triplicates. Cells were pretreated for 1 h with 80 μM ELP and ELP-P10, and GCV (control) or vehicle-treated, then infected with MCMV at an MOI of 3.0 or mock-infected. At 3 dpi, the CMC (carboxymethylcellulose) + DMEM overlay was removed. The cells were then harvested by trypsinization to determine the cell viability using trypan blue exclusion with a TC20 automated cell counter (BioRad Laboratories, Hercules, CA, USA) following the manufacturer’s protocol. Cell viability was determined using CellTiter 96® AQueous One Solution Cell Proliferation Assay (MTS). Breifly, MEFs were plated in 96 well plate and allowed to grow for 24 h followed by pretreatment with desired concentrations of ELP, ELP-P10 and GCV for 1 h, and then infected with MCMV at MOI of 3.0 or mock-infected. The cells were washed twice with PBS and overlayed with CMC + DMEM and incubated for 3 days. At 3 dpi, the CMC + DMEM overlay was removed, followed by addition of Complete DMEM and MTS reagent in a ratio of 5:1. The plates were incubated at 37 °C with 5% CO_2_ for 1–1.5 h. Absorbance was measured at 490 nm using Tecan's Magellan Microplate Reader. The percentage of cell survival was plotted for different treatment groups, and data were analyzed by one-way ANOVA using Tukey post hoc test.

### Rhodamine labeling of ELP-P10

ELP-P10 was fluorescently labeled on a unique cysteine residue using tetramethylrhodamine-5-maleimide. ELP-P10 was diluted to 100 µM in sterile PBS. The diluted protein was then mixed with 1 mM TCEP (tris-(2-carboxyethyl) phosphine) in Na_2_H_2_PO_4_ buffer for 1 h while rotating at room temperature followed by the addition of rhodamine to a final concentration of 200 μM. This mix was incubated with continuous stirring overnight at room temperature to ensure the labeling of protein with rhodamine. Any remaining free dye was removed by thermal cycling. Protein concentration and efficiency of labeling on the single cysteine residue were assessed by UV–Visible spectrophotometry as previously described^45^.

### Mice

All animal studies were conducted in accordance with the ARRIVE guidelines and the protocols approved by the Institutional Animal Care and Use Committee at the University of Mississippi Medical Center. All experiments were performed in accordance with relevant guidelines and regulations. Male and female Balb/c mice (6–9 weeks of age), maintained under specific pathogen-free conditions, were used for this study.

### Pharmacokinetics and biodistribution analysis of peptides

Mice (female, n = 4 per group) were anesthetized with isoflurane and administered 500 nmol/Kg of rhodamine-labeled ELP-P10/P10 by subcutaneous injection. Blood samples were collected from the tail vein at different time points and centrifuged to collect plasma. The fluorescence of plasma samples was directly measured using a Take 3 Microvolume Plate, measured using an Agilent Cytation 7 (Excitation: 535, Emission: 580, Gain: 100), and fit to a standard curve of rhodamine-labeled ELP and ELP-P10. For the biodistribution, a separate cohort of mice (female, n = 4 per group) were euthanized four hours after the injection with proteins or saline control, and the brain, spleen, liver, lungs, kidneys, and heart were harvested and assessed by ex-vivo whole organ imaging using the In Vivo Imaging System (IVIS, Perkin Elmer). The average radiant efficiency of each organ was adjusted by subtracting the autofluorescence values obtained from imaging the organs of an animal injected with saline and fit to a standard curve of known concentrations of ELP or ELP-P10. To determine pharmacokinetic parameters, plasma concentrations during the clearance phase (from the peak of the plasma concentration curve) were fit using a two-compartment pharmacokinetic model as described previously using GraphPad Prism^32^.

### In-vivo analysis of peptide efficacy

Mice (3 males, 3 females per treatment group) were anesthetized with isoflurane and subcutaneously injected with 1000 nmol/Kg of the P10, ELP, ELP-P10, or vehicle-treated 1 h prior to infection. One-hour post-treatment, mice were infected intraperitoneally with 1 × 10^6^ PFU of MCMV K181. Animals were treated with the same dose of peptides every 24 h, and their weight was monitored. Three days post-infection, animals were euthanized, and the liver, kidneys, spleen, heart were harvested and stored at − 80 °C. Virus titers in harvested organs were determined by plaque assay.

### Viral titers by plaque assay

MEFs were used for the plaque assays. MCMV-infected samples were harvested and stored at − 80 °C. The harvested organs were thawed at 37 °C and then stored on ice. The organs were then sonicated in PBS three times for 10 s each with a 10-s gap. Monolayers of MEFs were grown in 12 well tissue culture plates, and serial dilutions (10^–1^ to 10^–3^) of sonicated samples were absorbed on them for 2 h at 37 °C in 5% CO_2_ in triplicates. After 2 h, the sonicate was removed by suction followed by two washes with PBS. Then, pre-warmed CMC dissolved in complete DMEM was added and incubated for 3 days. At the endpoint, the CMC media was removed, cells were washed 2 times with PBS and fixed with 100% methanol for 7 min. Finally, the monolayers were stained with crystal violet for 30 min, then washed with tap water, air dried, and MCMV plaques were quantified.

### Statistics

Results from all experiments are expressed as mean ± SD or otherwise stated. Comparisons among three or more groups were made using one-way ANOVA or two-way ANOVA, as appropriate, in GraphPad Prism 9.0 (GraphPad Prism version 9.0.0, GraphPad Software, San Diego, CA, SA, access date 10 November 2021) with Tukey’s post-hoc tests (comparing mean of treatment groups to the vehicle) to correct for multiple comparisons. Differences between or among groups were considered significant at a *p* value of < 0.05.

### Supplementary Information

Supplementary Figure S1.

## Data Availability

All the data are included in the manuscript itself.
