# Supplementary material for: An Elastin-like Polypeptide-fusion peptide targeting capsid-tegument interface as an antiviral against cytomegalovirus infection
Source: Sci Rep. 2024 May 4;14:10253. doi: 10.1038/s41598-024-60691-6 (PMC11069587; doi:10.1038/s41598-024-60691-6)
Supplement: Supplementary file 1 — Supplementary Figure S1. [file 41598_2024_60691_MOESM1_ESM.docx]

**Title:** An ELP-fusion Peptide Targeting Capsid-Tegument Interface as an Antiviral against Cytomegalovirus Infection

**Authors:** Komal Beeton^1^, Dipanwita Mitra^1^, Adesanya A. Akinleye^2^, John A. Howell^3^, Christian S. Yu^1^, Gene L. Bidwell III^2,3^, and Ritesh Tandon^1,4#*^.

**Affiliations**

^1^Center for Immunology and Microbial Research, Department of Cell and Molecular Biology, University of Mississippi Medical Center, 2500 North State Street, Jackson, MS 39216, USA.

^2^Department of Pharmacology and Toxicology, University of Mississippi Medical Center, 2500 North State Street, Jackson, MS 39216, USA

^3^Department of Neurology, University of Mississippi Medical Center, 2500 North State Street, Jackson, MS 39216, USA

^4#^Office of Research Infrastructure Programs, National Institute of Health, 6701 Democracy Blvd., Bethesda, MD 20892

* Correspondence: ritesh.tandon@nih.gov; Tel.: (301-594-5304). #This article was prepared while Ritesh Tandon was employed at the University of Mississippi Medical Center. The opinions expressed in this article are author’s own and do not reflect the view of the National Institutes of Health, the Department of Health and Human Services, or the United States Government.

**ELP-P10 protects MCMV infected cells from virus induced lytic cell death**

1. **b)**

**Supplementary Figure S1: Cell Viability (%) in (a) Infected MEFs (b) Uninfected MEFs with different treatments groups.** MEFs were pretreated with ELP and ELP-P10, and GCV (control) or vehicle-treated for 1 hour in triplicates, and then infected with a high MOI of 3.0 with MCMV K181 or vehicle-infected. At 3 dpi CMC + DMEM was removed followed by addition of Complete DMEM and MTS reagent in a ratio of 5:1 and absorbance were measured at 490nm using Tecan's Magellan Microplate Reader. The percentage of cell survival was plotted for different treatment groups and data was analyzed by one-way Anova using Tukey post hoc test.
